# Proteomic analysis of the signaling pathway mediated by the heterotrimeric Gα protein Pga1 of *Penicillium chrysogenum*

**DOI:** 10.1186/s12934-016-0564-x

**Published:** 2016-10-06

**Authors:** Ulises Carrasco-Navarro, Rosario Vera-Estrella, Bronwyn J. Barkla, Eduardo Zúñiga-León, Horacio Reyes-Vivas, Francisco J. Fernández, Francisco Fierro

**Affiliations:** 1 Departamento de Biotecnología, Universidad Autónoma Metropolitana-Unidad Iztapalapa, México D.F., México; 2 Instituto de Biotecnología, Universidad Nacional Autónoma de México, Cuernavaca, Morelos México; 3 Southern Cross Plant Science, Southern Cross University, Lismore, NSW Australia; 4 Laboratorio de Bioquímica-Genética, Instituto Nacional de Pediatría, Secretaría de Salud, México D.F., México

**Keywords:** *Penicillium chrysogenum*, Pga1 Gα subunit, Signal transduction, Heterotrimeric G protein, Proteomics, Penicillin biosynthesis

## Abstract

**Background:**

The heterotrimeric Gα protein Pga1-mediated signaling pathway regulates the entire developmental program in *Penicillium chrysogenum*, from spore germination to the formation of conidia. In addition it participates in the regulation of penicillin biosynthesis. We aimed to advance the understanding of this key signaling pathway using a proteomics approach, a powerful tool to identify effectors participating in signal transduction pathways.

**Results:**

*Penicillium chrysogenum* mutants with different levels of activity of the Pga1-mediated signaling pathway were used to perform comparative proteomic analyses by 2D-DIGE and LC–MS/MS. Thirty proteins were identified which showed differences in abundance dependent on Pga1 activity level. By modifying the intracellular levels of cAMP we could establish cAMP-dependent and cAMP-independent pathways in Pga1-mediated signaling. Pga1 was shown to regulate abundance of enzymes in primary metabolic pathways involved in ATP, NADPH and cysteine biosynthesis, compounds that are needed for high levels of penicillin production. An in vivo phosphorylated protein containing a pleckstrin homology domain was identified; this protein is a candidate for signal transduction activity. Proteins with possible roles in purine metabolism, protein folding, stress response and morphogenesis were also identified whose abundance was regulated by Pga1 signaling.

**Conclusions:**

Thirty proteins whose abundance was regulated by the Pga1-mediated signaling pathway were identified. These proteins are involved in primary metabolism, stress response, development and signal transduction. A model describing the pathways through which Pga1 signaling regulates different cellular processes is proposed.

**Electronic supplementary material:**

The online version of this article (doi:10.1186/s12934-016-0564-x) contains supplementary material, which is available to authorized users.

## Background

The phylum Ascomycota includes many species of biotechnological importance. Conidial germination, extension of hyphae and sporulation constitute the main processes of the growth/development program of these fungi. These processes are carried out by specialized morphogenic machinery, coordinated and regulated by mechanisms that are still being elucidated. Ascomycetous fungi are also lavish producers of secondary metabolites, many of which are of great importance for medical use and other applications [1]. Development and secondary metabolism are often subjected to coordinated regulation in ascomycetous fungi through cellular signaling processes [2]. Heterotrimeric G proteins mediate various important cellular processes in fungi in response to environmental stimuli [3]. The Gα subunits of fungal heterotrimeric G proteins are classified in three subgroups (I–III) [4], of which, subgroup I has been shown to participate in the regulation of several relevant biological processes, such as germination, growth, asexual development, pathogenicity and secondary metabolism [3].

*Penicillium chrysogenum* has had a key historical role in the development of industrial microbiology, and is currently one of the most important species in the biotechnological industry as producer of penicillin [5] and other β-lactam antibiotic precursors [6]. The *P. chrysogenum* heterotrimeric Gα protein Pga1, belonging to the fungal subgroup I, has been characterized and shown to participate in the regulation of the global developmental program of the fungus, from spore germination to conidia formation [7–9]. In addition, it participates in the regulation of penicillin biosynthesis [10]. Characterizing and understanding the function of the Pga1-mediated signal transduction pathway in *P. chrysogenum* is therefore of great interest, and will serve as a model for other subgroup I heterotrimeric Gα protein-mediated signaling pathways in ascomycetous filamentous fungi. The current knowledge on the molecular mechanisms through which this pathway regulates development, secondary metabolism and other processes in fungi is still limited. A well-established downstream effector of subgroup I Gα subunits is adenylyl cyclase, which synthesizes cAMP resulting in the activation of the protein kinase A (PKA) [8, 9, 11–15]. Nevertheless, additional and yet uncharacterized cAMP-independent pathways for Pga1 signaling are present in *P. chrysogenum* [8].

Proteomic analysis is a powerful tool to study signaling pathways and identify involved effectors [16, 17]. In general the correlation between mRNA levels and protein levels has been shown to be low [18, 19], therefore proteomic approaches are an important source of information that can complement the data obtained by transcriptomics. Proteomics has been used, for instance, to elucidate the metabolic effects caused by the deletion of specific genes in fungi [20]. In this study, we aimed to identify effectors of the Pga1-mediated signaling pathway using strains with different levels of Pga1 activity, and thus gain insight into the mechanisms through which Pga1 transduces signals and regulates the cell response to stimuli.

## Results and discussion

### Detection and identification of proteins showing differential expression dependent on Pga1 activity

**Fig. 1:**
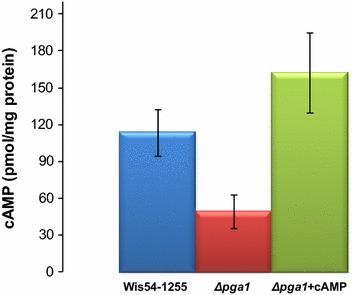
Intracellular cAMP concentration in strains Wis54-1255 and Δ*pga1*, and in the condition Δ*pga1* + cAMP, after 72 h of cultivation in flasks (250 rpm and 25 °C) with PMMY medium

We used a 2D-DIGE approach to detect proteins which showed higher or lower abundance in pairwise comparisons between strains with different Pga1 activity (see “Methods” section, Table 3). Strains Wis54-1255, PgaG42Rpyr-T and ∆*pga1* [8] were used for the comparisons, plus a fourth additional condition: strain ∆*pga1* + cAMP, with db-cAMP and theophylline added to the culture medium to increase intracellular cAMP levels [8]. The intracellular cAMP concentration in strain Wis54-1255 and in strain ∆*pga1* with and without addition of db-cAMP and theophylline is shown in Fig. 1. The condition ∆*pga1* + cAMP was used to distinguish between cAMP-mediated and cAMP-independent Pga1 signaling; in previous work we found that Pga1 causes an increase in the intracellular cAMP level [8, 9], but some Pga1-regulated processes, such as conidiation, are mostly cAMP-independent [8].

**Fig. 2:**
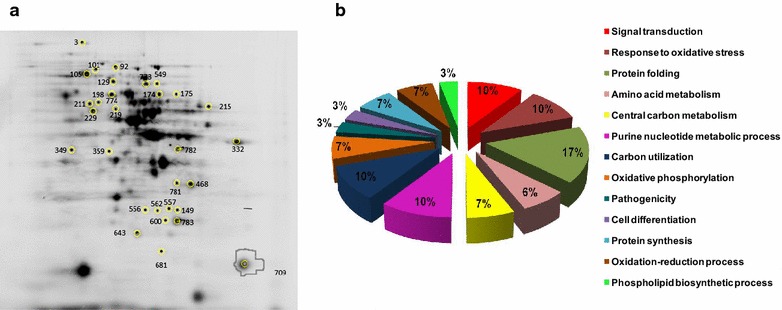
**a** 2D-DIGE dye overlay gel image (see Table 4 for sample composition). The first dimension was performed using a 17 cm IPG strip, and the second dimension using a 12 % linear acrylamide gel. *Spots* that showed changes in abundance are* numbered* and* encircled*; the position numbers correspond to the spot numbers listed in Table 1. The relative changes in normalized spot volume of protein amount were assessed by biological variation (BVA) across all the DIGE experiments. **b** Classification of the identified proteins according to their biological function, using the NCBI, Uniprot and KEEG databases and bibliographic search

A total of eight gels were run following an experimental scheme previously reported by Vera-Estrella et al. [21] (see “Methods”, Table 4). After analysis with DeCyder Software, a total of 30 spots were detected which showed significant changes in abundance of >1.5 (p ≤ 0.05 in ANOVA test) in one or more of the pairwise comparisons between strains/conditions (Fig. 2a). These spots were excised from the gels, analyzed by LC–MS/MS and identified by Mascot (see “Methods” section). The identified proteins are summarized in Table 1 (see also Additional file 1: Table S1).

**Table 1:** Identified proteins showing significant changes of abundance in strains with different Pga1 activity

#Spot	Protein	Name	Function
101	Pc12g05640	Strong similarity to heat shock protein sspB—*Aspergillus nige*r (HSP90)	Protein folding
105	Pc22g11240	HSP70 (nucleotide binding domain), strong similarity to heat shock protein 70 HSP70—*Ajellomyces capsulatus*	Protein folding
129	Pc22g10220	Strong similarity to dnaK-type molecular chaperone Ssb2—*Saccharomyces cerevisiae*	Protein folding
774	Pc16g11070	Strong similarity to mitochondrial heat shock protein Hsp60 - *Saccharomyces cerevisiae*	Protein folding
681	Pc22g19060	Strong similarity to cyclophilin cypB—*Aspergillus nidulans*	Protein folding
3	Pc22g05690	Strong similarity to hypothetical protein contig12.tfa_1730cg—*Aspergillus fumigatus* [Protein containing PH domain (pleckstrin homology domain)]	Signal transduction
198	Pc22g17420	Strong similarity to hypothetical protein contig_1_153_scaffold_12.tfa_500cg—*Aspergillus nidulans* (Protein containing ankyrin repeat and von Willebrand factor type A (vWFA) domains)	Signal transduction
562	Pc22g01260	Strong similarity to small G-protein Gsp1—*Candida albicans* (Ran small GTPase)	Signal transduction
92	Pc16g11860	Strong similarity to catalase R catR—*Aspergillus niger*	Response to oxidative stress
600	Pc22g25220	Strong similarity to 1,4-benzoquinone reductase qr—*Phanerochaete chrysosporium*	Response to oxidative stress
643	Pc22g25220	Strong similarity to 1,4-benzoquinone reductase qr—*Phanerochaete chrysosporium*	Response to oxidative stress
175	Pc18g05320	Strong similarity to IMP dehydrogenase IMH3—*Candida albicans* (inosine monophosphate dehydrogenase)	Purine nucleotide metabolic process
468	Pc22g20960	Strong similarity to urate oxidase uaz—*Aspergillus flavus*	Purine nucleotide metabolic process
709	Pc22g19100	Strong similarity to 5-aminoimidazole-4-carboxamide ribotide transformylase Ade17—*Saccharomyces cerevisiae*	Purine nucleotide metabolic process
211	Pc21g12590	Similarity to 6-hydroxy-d-nicotine oxidase 6-HDNO—*Arthrobacter oxidans*	Carbon utilization
549	Pc22g24530	Similarity to hypothetical protein MDB19—*Arabidopsis thaliana* (Dienelactone hydrolase)	Carbon utilization
557	Pc21g10590	Strong similarity to carbonic anhydrase pca1—*Porphyridium purpureum*	Carbon utilization
149	Pc20g07710	Sulfate adenylyltransferase	Amino acid metabolism
332	Pc22g13130	Strong similarity to mitochondrial aspartate aminotransferase mAspAT—*Mus musculus*	Amino acid metabolism
174	Pc18g06000	Strong similarity to pyruvate kinase pkiA—*Aspergillus niger*	Central carbon metabolism
773	Pc13g12450	Strong similarity to transketolase Tkl1—*Saccharomyces cerevisiae*	Central carbon metabolism
215	Pc12g03370	Strong similarity to mitochondrial F1-ATPase alpha-subunit Atp1—*Saccharomyces cerevisiae*	Oxidative phosphorylation
229	Pc21g10070	Strong similarity to H^+^-transporting ATP synthase Beta chain—*Neurospora crassa*	Oxidative phosphorylation
556	Pc20g05750	Strong similarity to levodione reductase like protein An03g05050—*Aspergillus niger* (short chain dehydrogenase)	Oxidation–reduction process
782	Pc13g07960	Strong similarity to alcohol dehydrogenase ADH like protein An04g02690—*Aspergillus niger*	Oxidation–reduction process
781	Pc18g02110	Strong similarity to hypothetical protein contig1471_1.tfa_1240 wg—*Aspergillus fumigatus* (Ribosomal protein S3)	Protein synthesis
783	Pc16g10560	Strong similarity to cytoplasmic ribosomal protein of the large subunit L10—*Saccharomyces cerevisiae*	Protein synthesis
349	Pc13g09680	Strong similarity to aspartyl proteinase candidapepsin—*Candida albicans*	Pathogenicity
359	Pc13g04170	Similarity to cell polarity protein tea1p—*Schizosaccharomyces pombe*	Cell differentiation
219	Pc22g04840	Strong similarity to hypothetical phosphatidyl synthase SPAC22A12.08c—*Schizosaccharomyces pombe*	Phospholipid biosynthetic process

### Classification of the identified proteins according to their biological function

Using the NCBI, Uniprot and KEGG databases, as well as bibliographical search, we classified the identified differentially expressed proteins in 13 groups defined by their biological function (Fig. 2b; Table 1). Proteins involved in protein folding were the most numerous, and additionally two ribosomal proteins were identified, suggesting an important role for cellular protein synthesis in the processes regulated by the Pga1-mediated pathway. Three proteins related to signal transduction, response to oxidative stress and purine metabolism, respectively, were also shown to be significantly altered in abundance. Identification of two proteins belonging to carbohydrate catabolic pathways and two subunits of the ATP synthase indicates that energy metabolism is also important in Pga1-regulated cellular processes. In addition, we found proteins involved in amino acid metabolism, carbon utilization, oxidation–reduction processes, cell differentiation and phospholipid biosynthesis.

**Fig. 3:**
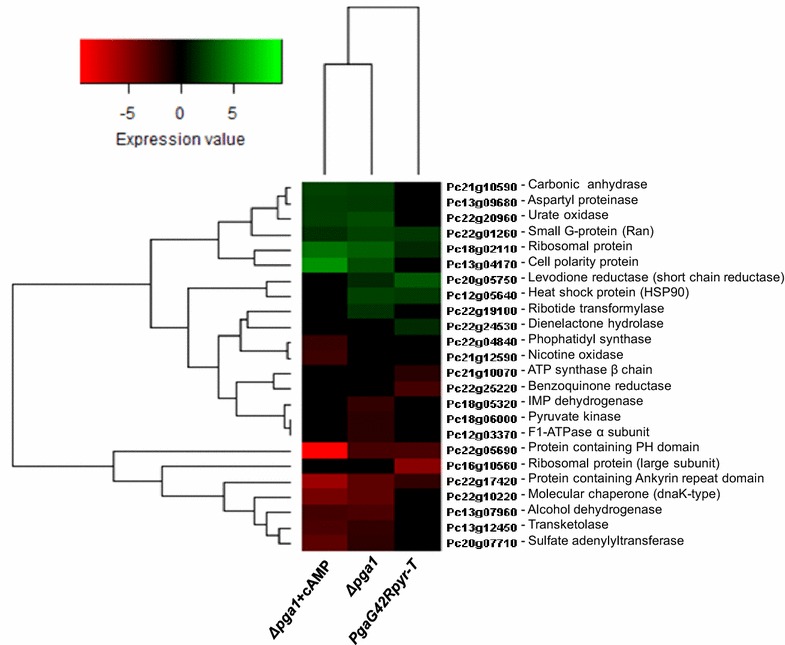
Heat map showing the changes in protein abundance between strains PgaG42Rpyr-T, ∆*pga1* and condition ∆*pga1* + cAMP with respect to the Wis-54-1255 strain (wild type Pga1). The neutral (*brown*) colour indicates that no significant change occurred between a given strain/condition with respect to strain Wis54-1255, whereas *red* and *green* colours indicate lower and higher abundance, respectively. The Heat map was generated with the program R. The complete names of the proteins are provided in Table 1

**Fig. 4:**
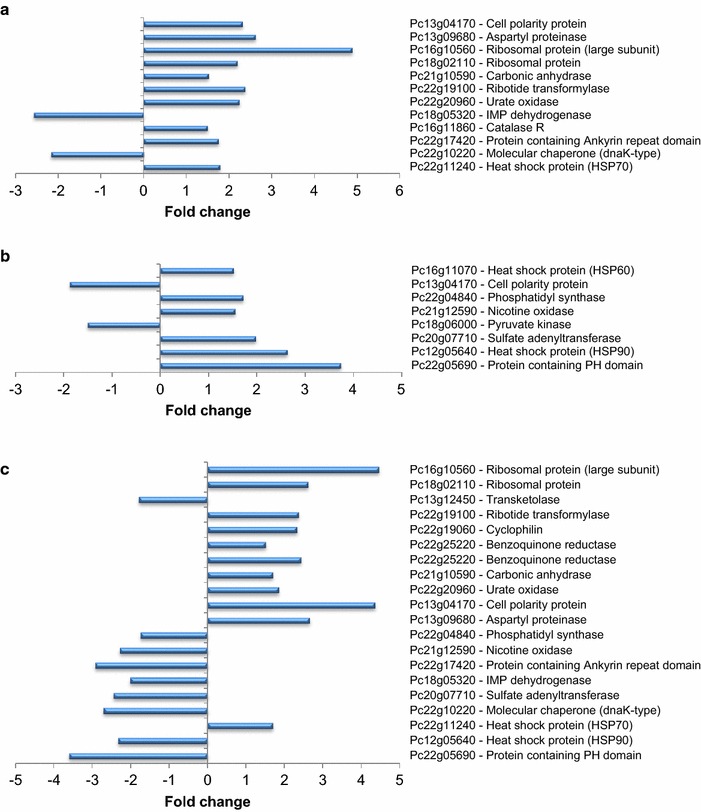
Changes in abundance of proteins in pairwise comparisons between strains/conditions: Δ*pga1* vs. PgaG42Rpyr-T (**a**), Δ*pga1* vs. Δ*pga1* + cAMP (**b**), and Δ*pga1* + cAMP vs. PgaG42Rpyr-T (**c**). The complete names of the proteins are provided in Table 1

The identification of proteins within these categories suggests that primary metabolism is strongly affected by Pga1 signaling, along with protein synthesis/folding. In the next sections we will discuss the significance of these findings and how the identified proteins may relate to Pga1-regulated processes. Most of the identified proteins showed differences in abundance between strain Wis54-1255 and one or more of strains/condition PgaG42Rpyr-T, ∆*pga1* or ∆*pga1* + cAMP (Fig. 3), and some showed differences only between the three latter (Fig. 4).

### Regulation of the central metabolism and oxidative phosphorylation by Pga1 signaling

Pyruvate kinase (Pc18g06000), a key regulator of glycolysis, the mitochondrial F1-ATPase alpha-subunit Atp1 (Pc12g03370), which participates in oxidative phosphorylation, and a transketolase (Pc13g12450), which participates in the non-oxidative phase of the pentose phosphate pathway, are in low abundance in the absence of Pga1 (strain ∆*pga1*). Pyruvate kinase and Atp1 abundance is restored to wild type levels when the cAMP concentration is increased (condition ∆*pga1* + cAMP), but transketolase abundance remains low also in this condition (Fig. 3), indicating that pyruvate kinase and Atp1 abundance is controlled by Pga1 via cAMP/PKA, whereas the regulation of transketolase by Pga1 is cAMP-independent.

Furthermore, pyruvate kinase may also be phosphorylated by PKA, since it contains two putative PKA phosphorylation sites (consensus RRXS) in its amino acid sequence: at Ser 36 (RRTS) and Ser 237 (RRGS), with a 1.71 and 0.97 score, respectively, in the prediction of phosphorylation calculated by pkaPS server [22]. It has been demonstrated that pyruvate kinase Pyk1 of *Saccharomyces cerevisiae* is phosphorylated in vivo and in vitro by PKA [23, 24].

Pga1 prevents premature conidiation, as inferred from the hyperconidiating phenotype of strain ∆*pga1* [8]. According to the abundance of the three proteins mentioned above, it seems that energy metabolism (glycolysis and pentose phosphate pathways and oxidative phosphorylation) slows down as part of the developmental process of conidiophore and conidia formation, which in turn involves the slowdown and termination of the apical growth of hyphae.

Another component of the mitochondrial ATPase, the H^+^-transporting ATP synthase β-chain (Pc21g10070), is present in lower amounts in the PgaG42Rpyr-T strain than in the ∆*pga1* and Wis54-1255 strains (Fig. 3). Yuan and Douglas [25] demonstrated that *S. cerevisiae* mutants lacking the F1-ATPase alpha-subunit Atp1 exhibit delayed kinetics of protein import for several mitochondrial precursors, among them the F1β-subunit, which accumulates as a translocation intermediate in absence of Atp1. It will thus be interesting to determine if *P. chrysogenum* Atp1 (Pc12g03370) has a similar function regulating the entry of constituents of the electron transport chain and the ATP synthase into the mitochondria, thus regulating the oxidative phosphorylation process, which would be controlled upstream by the Pga1 signaling pathway.

Conidia germination is controlled by Pga1 via cAMP in response to carbon sources [9]. Accordingly the Δ*pga1* strain, whose cAMP levels are lower than in strains with a functional Pga1 (Fig. 1) [8], shows delayed germination and lower germination efficiency [9]. The Pga1-mediated signaling pathway senses glucose and other carbon sources to trigger germination [9], and the glycolysis and oxidative phosphorylation rates are presumed to be lower in the absence of Pga1, an effect that is reversed by the induced increase of intracellular cAMP, as inferred from the abundance levels of pyruvate kinase and the F1-ATPase alpha-subunit Atp1 (discussed above). This suggests that Pga1 signaling links glucose sensing to regulation of sugar catabolic pathways and oxidative phosphorylation. The absence of Pga1 in strain ∆*pga1* results in a reduced ability to sense glucose, hence enzymes and proteins participating in energy metabolism are in lower abundance in this strain. Glucose limitation has been shown to cause a decrease in the expression of genes of the glycolytic pathway in *Neurospora crassa* [26]. All these possible effects have to be further investigated in *P. chrysogenum*.

### Pga1 signaling and penicillin production

Decreases of penicillin yields in strains lacking Pga1 activity and increases in strains expressing a constitutively active Pga1 have been previously reported [10]. Transketolase was less abundant in strain Δ*pga1* than in strains Wis54-1255 and PgaG42Rpyr-T (Figs. 3, 4). This enzyme is part of the pentose phosphate pathway, the main source of the reduced form of the NADP^+^ coenzyme (NADPH). Previous studies described that penicillin production requires high NADPH concentrations [27], and that transketolase and other enzymes of the pentose phosphate pathway are overexpressed in the high yield producer strain AS-P-78 [28]. The high demand for NADPH is due to its requirement for the biosynthesis of penicillin precursors, such as the amino acids valine and cysteine [29]. The low abundance of transketolase in strain Δ*pga1* would reduce the pentose phosphate pathway flux rate, affecting NADPH formation, which in turn would contribute to the low penicillin production in this strain. Transketolase abundance is not recovered by restoring normal cAMP levels in strain Δ*pga1* (i.e., transketolase regulation by Pga1 signaling is cAMP-independent), which agrees with the previously reported lack of effect of induced high cAMP levels on penicillin production [10].

Penicillin production also demands high levels of ATP [30], which is mainly due to the high ATP requirement of the enzyme δ-(l-α-aminoadipyl)-l-cysteinyl-d-valine synthetase, which catalyzes the first step of penicillin biosynthesis [31]. The ∆*pga1* strain is probably inefficient in ATP production in comparison to strains with a functional Pga1 due to the lower abundance of the F1-ATPase alpha-subunit Atp1 (see above), which would affect penicillin production in this strain. Interestingly, the inducer of penicillin biosynthesis 1,3-diaminopropane also causes an increase in the abundance of the F1-ATPase alpha-subunit Atp1 when added to cultures of the Wis54-1255 strain, as observed in a comparative proteomics study [32].

Amino acid metabolism is also affected by Pga1 signaling. Of particular relevance is the abundance of the sulfate adenylyltransferase (Pc20g07710), which is lower in strain ∆*pga1* (−1.7-fold), and even more so in condition ∆*pga1* + cAMP (−3.8-fold), than in the strains with a functional Pga1 (Figs. 3, 4). This enzyme is involved in the biosynthesis of cysteine, one of the three amino acids precursors of penicillin biosynthesis, which is a substrate of the δ-(l-α-aminoadipyl)-l-cysteinyl-d-valine synthetase and required in large quantities for efficient penicillin production [30]. In strain DS17690, a very high yield penicillin producer, the genes encoding enzymes of the cysteine biosynthetic pathway were upregulated [33], among them the sulfate adenylyltransferase gene. Similarly, the AS-P-78 strain, a high yield penicillin producer, showed higher abundance of proteins related to cysteine biosynthesis than the wild type NRRL 1951 and the Wis54-1255 strains [28]. The decreased expression of the sulfate adenylyltransferase in strain ∆*pga1* likely contributes to its lower penicillin production [10]. The induced increase of cAMP decreases the abundance of sulfate adenylyltransferase (Fig. 3), and interestingly cAMP concentration in penicillin non-producing conditions was found to be higher than in producing conditions in a controlled steady-state flux chemostat culture [27]. All evidence suggests that penicillin production is not regulated or is even negatively regulated by cAMP.

According to a metabolome study of a high yield industrial strain in penicillin producing and non-producing conditions [30] and to a proteomics study of low, intermediate and high yield penicillin producing strains [28], the main features of primary metabolism that have a major impact on penicillin production are: (1) high cysteine, but not valine or α-aminoadipate, availability, (2) high NADPH supply, and (3) high ATP supply. In this study, we have observed that Pga1 has a role in all these processes, regulating the expression of proteins related to the biosynthesis of cysteine, NADPH and ATP. Therefore, we conclude that Pga1 signaling is an important regulator of the primary metabolism processes that lead to penicillin biosynthesis.

A dnaK-type molecular chaperone (Pc22g10220), similar to the *S. cerevisiae* Ssb2, is less abundant in strain ∆*pga1* (independently of cAMP) than in strains Wis54-1255 and PgaG42Rpyr-T. Interestingly, this protein has been shown to increase its abundance in the presence of 1,3-diaminopropane [32]. Therefore, this chaperone is more abundant in conditions that stimulate penicillin production, i.e. when there is a functional Pga1 and in the presence of 1,3-diaminopropane. The Ssb2 protein participates in glucose sensing in *S. cerevisiae*. Ssb2 is required to maintain the Snf1 protein kinase in a dephosphorylated, and thus inactive, form [34]. The Snf1 protein kinase allows expression of genes involved in the utilization of alternative carbon sources in the absence of glucose by inhibiting the action of the repressing complex Mig1/Ssn6/Tup1 [35], which is responsible for carbon catabolite repression. In *P. chrysogenum* the penicillin genes are subject to carbon catabolite repression which is exerted by the transcription factor CreA [36]. It will therefore be of great interest to study the possible involvement of Pc22g10220 in glucose sensing and carbon catabolite repression, and analyze if the regulation of the penicillin biosynthetic genes by Pga1 [10] is mediated by this chaperone and CreA.

A putative dienelactone hydrolase (Pc22g24530) is more abundant in strain PgaG42Rpyr-T than in strain Wis54-1255 (1.63-fold). This protein was also reported to be more abundant in the high yield penicillin producer AS-P-78 in comparison with lower producing strains [28]. Strains expressing a constitutively active Pga1 Gα subunit (Pga1^G42R^) produce higher amounts of penicillin than strains with a wild type Pga1 [10]. The significance of these findings and the possible relation of dienelactone hydrolase to penicillin production are still unclear.

### Pga1 signaling regulates proteins involved in purine metabolism

Deletion of the *pga1* gene results in lower abundance of an inosine monophosphate (IMP) dehydrogenase (Pc18g05320) (−1.89-fold). This enzyme catalyzes the conversion of IMP to xanthosine monophosphate (XMP) in the guanosine monophosphate (GMP) biosynthetic pathway, which would result in lower production of GTP. We can interpret this result in the same sense as those described above for pyruvate kinase and the F1-ATPase alpha-subunit Atp1. The three proteins show an identical pattern of abundance between strains/conditions: a decrease in the absence of Pga1 (∆*pga1* strain) and recovery to wild type levels when the cAMP concentration is raised (condition ∆*pga1* + cAMP). Absence of Pga1 causes premature and extensive conidiation, concomitant with a cessation of vegetative growth of the hyphae [8]. In this situation, energy metabolism should slow down, and this includes GTP production. Increasing cAMP concentration in the ∆*pga1* strain results in an increase in abundance of the three proteins (Fig. 3), an effect that may be related to the decrease of conidiation in strain ∆*pga1* when cAMP intracellular levels are increased [8]. In *Stagonospora nodorum*, deletion of the *gna1* gene causes an increase of the GMP synthase [37], which would result in the higher production of GTP. However, the role of the Gna1 Gα subunit in asexual sporulation is opposite to that of Pga1; whereas Pga1 represses conidiation, Gna1 is essential for sporulation in *S. nodorum*.

Two proteins involved in purine metabolism were more abundant in strain ∆*pga1*, a 5-aminoimidazole-4-carboxamide ribotide transformylase (Pc22g19100) and a urate oxidase, or uricase (Pc22g20960) (2.84-fold). The latter was also increased in the Δ*pga1* + cAMP condition (2.34-fold), indicating that its regulation by Pga1 is cAMP-independent. In *Magnaporthe oryzae* an uricase and other proteins involved in the metabolism of nucleotides showed changes in abundance when genes encoding RGS were deleted [38]. These results indicate that G protein signaling regulates purine metabolism.

### Proteins involved in protein folding regulated by Pga1 signaling

We identified five proteins with a protein folding function and two more involved in protein synthesis whose expression is controlled by Pga1 signaling (Table 1). There appears to be no common pattern in the abundance of the proteins involved in protein folding. Strain ∆*pga1* shows higher resistance to several stress conditions [9], and therefore protein folding activities in general should be expected to increase in this strain, as observed for proteins Pc12g05640, Pc22g11240 and Pc22g19060, which show strong similarity to fungal HSP90, HSP70 and cyclophilin cypB, respectively. However, protein Pc22g10220, a putative dnaK-type molecular chaperone, is less abundant in strain ∆*pga1*. The higher abundance of the HSP70 chaperone (Pc22g11240) in strain ∆*pga1* could be related to the increased viability of conidia of this strain after a heat shock treatment [9].

### Pga1 signaling and oxidative stress response

Two proteins were identified with strong similarity to enzymes participating in oxidative stress response: a catalase R (Pc16g11860) and a benzoquinone reductase (Pc22g25220). In both cases protein abundance is higher when Pga1 activity is lower. This was an expected result, since inactivation or absence of Pga1 results in higher resistance to oxidative stress induced by H_2_O_2_ [9]. A similar increase in the abundance of proteins related to the oxidative stress response was found in the high yield penicillin producer strain AS-P-78 with respect to lower yield producers [28], but the physiological significance of this result remains unclear. In *S. nodorum*, a bifunctional catalase-peroxidase was found to be less abundant in a ∆*gna1* mutant than in the wild type [37]; however, ∆*gna1* mutants are unable to sporulate, a phenotype opposite to that of ∆*pga1* mutants in *P. chrysogenum*, and they do not show increased resistance or sensitivity to oxidative stress [37].

### Putative signal transduction mediators in Pga1 signaling

**Fig. 5:**
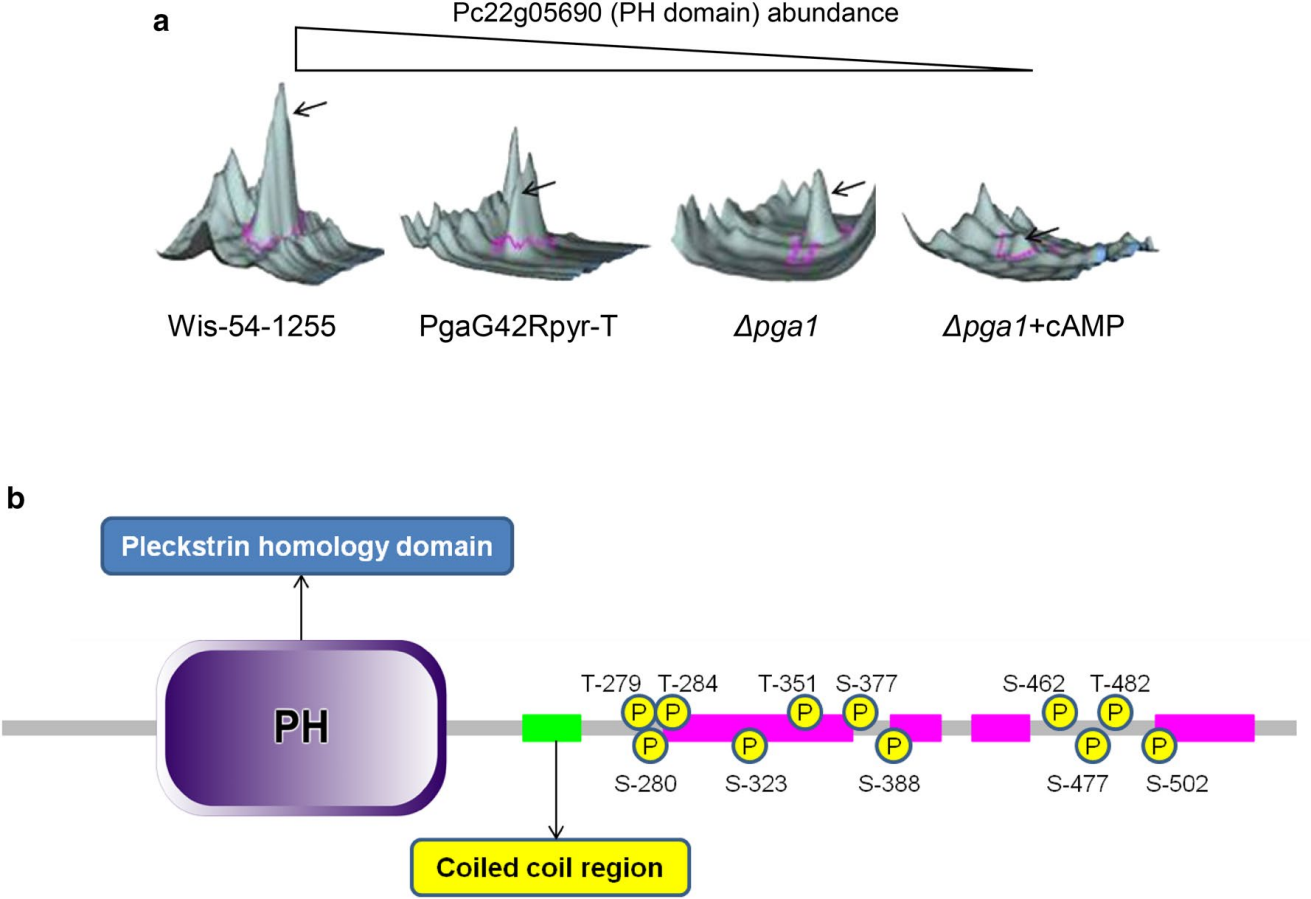
**a** Abundance of the Pc22g05690 protein in the different strains/conditions. The fluorescence signal was obtained with the program DeCyder 2-D differential analysis software. **b** Schematic representation of the Pc22g05690 protein showing the pleckstrin homology (PH) domain close to the N-terminal end, the coiled-coil region, and the eleven phosphorylated sites identified by LC–MS/MS. The domain structure of the protein was generated with SMART (simple modular architecture research tool, http://smart.embl-heidelberg.de)

A protein with a pleckstrin homology (PH) domain (Pc22g05690) was present in significantly lower abundance in both the ∆*pga1* strain (−2.57-fold) and the PgaG42Rpyr-T strain (−2.66-fold) with respect to strain Wis54-1255, and its abundance was further decreased by cAMP down to −9.6-fold in the condition Δ*pga1* + cAMP (Fig. 5a). Therefore the expression of this protein seems to be positively regulated by Pga1 in a cAMP-independent manner, and negatively regulated by high concentrations of cAMP. Proteins containing this domain have been linked to different functions, such as signal transduction and cytoskeleton organization, and it has been shown that G protein-coupled receptor kinases (GRKs) can interact with Gβγ dimers of heterotrimeric G proteins through their PH domain, causing an increase in phosphorylation of activated G protein-coupled receptors (GPCRs) [39, 40].

The Pc22g05690 protein is hyperphosphorylated in vivo (Fig. 5b), thus presenting a high degree of post-translational regulation characteristic of proteins involved in signal transduction [41]. From a total of eleven phosphorylation sites, five are putative targets for PKA, which is activated by cAMP and is part of the heterotrimeric G protein signaling pathway, and the remaining sites are putative targets for other protein kinases (Table 2).

**Table 2:** Prediction of the protein kinases targeting the phosphorylated sites identified by LC–MS/MS in protein Pc22g05690

Site	Kinase	Score	Phosphopeptides identified (LC–MS/MS)
T-279	PKA	0.64	(R)KRp**T**SIFGTLLGK(K)
S-280	PKA	0.64	(R)KRTp**S**IFGTLLGK(K) (K)RTp**S**IFGTLLGK(K) (R)Tp**S**IFGTLLGK(K)
T-284	PKC	0.85	(R)KRTSIFGp**T**LLGK(K)
S-323	–	–	(K)AAEPTTESp**S**AEAPAPVAAETAETAAAPTK(A)
T-351	CKII	0.55	(K)AAEPTAEp**T**PAETTEAAKEEAK(D)
S-377	PKA	0.78	(K)RAp**S**IFGNFFQK(V)
S-388	p38MAPK	0.56	(K)VAp**S**PSQEK(S) (K)VAp**S**PSQEKSEK(E)
S-462	PKA	0.8	(K)RRTp**S**FFGNLGMK(K)
S-477	CKII	0.66	(K)EKKADp**S**DNEVTDGEAK(E) (K)ADp**S**DNEVTDGEAK(E) (K)KADp**S**DNEVp**T**DGEAKETK(A)
T-482	CKII	0.72	(K)KADSDNEVp**T**DGEAKETK(A) (K)KADp**S**DNEVp**T**DGEAKETK(A)
S-502	PKA	0.69	(R)KPp**S**KAVKLDKEEVAAAETEAK(A)

The analysis was performed with the software NetPhosK 1.0 [55]

**Fig. 6:**
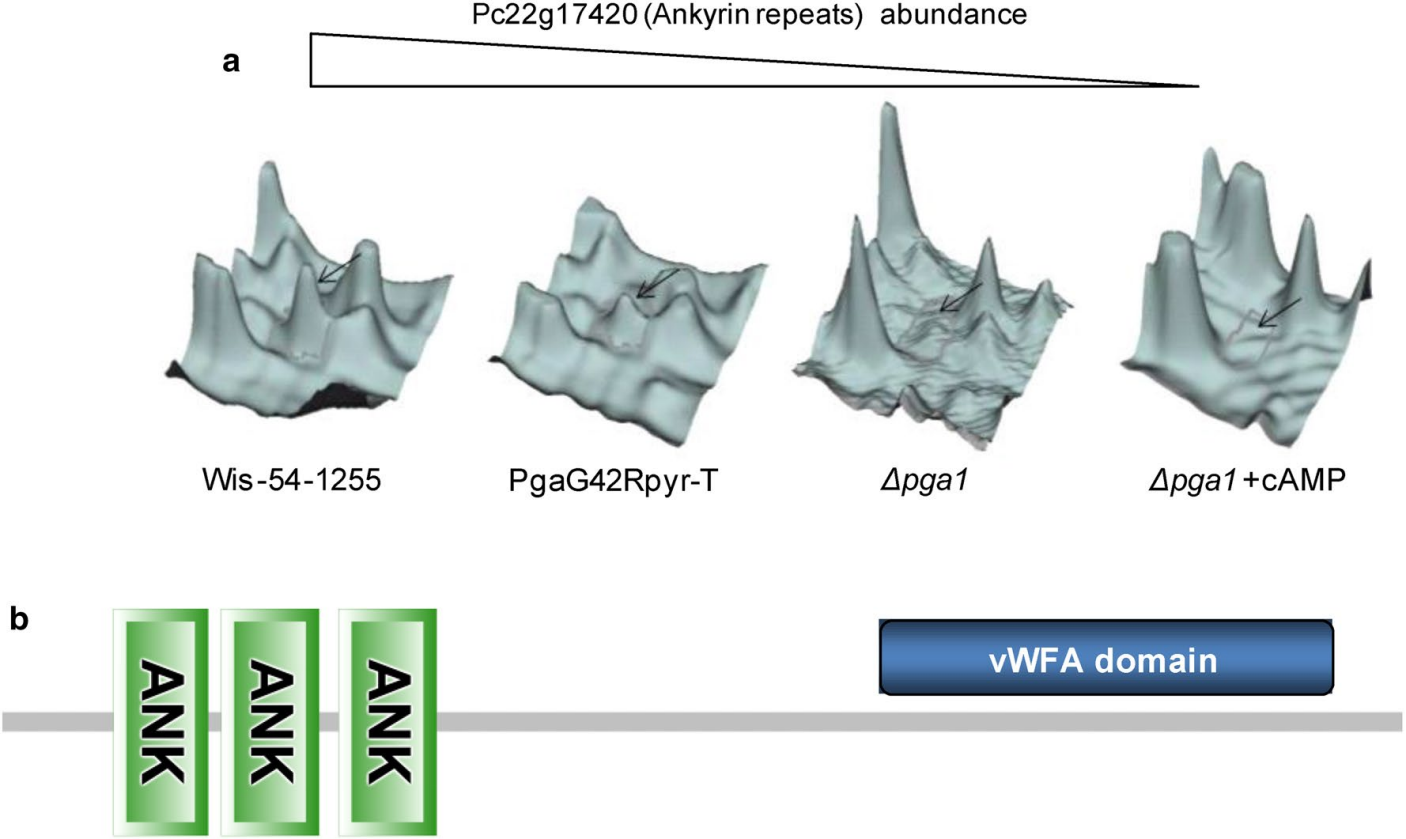
**a** Abundance of the Pc22g17420 protein in the different strains/conditions. The fluorescence signal was obtained with the program DeCyder 2-D differential analysis software. **b** Schematic representation of the Pc22g17420 protein, showing the ankyrin repeat domain close to the N-terminal end and the vWFA domain at the C-terminal region. The domain structure of the protein was generated with SMART (simple modular architecture research tool, http://smart.embl-heidelberg.de)

The Pc22g17420 protein, containing a domain of ankyrin repeats and a von Willebrand factor type A (vWFA) domain (Fig. 6b), shows a very similar pattern of expression to that of protein Pc22g05690 (Fig. 6a). Therefore, these two putative signal-transducing proteins are downregulated when Pga1 does not have a normal activity. The ankyrin repeat domain is involved in protein–protein interactions, and proteins containing this domain participate in processes such as cell cycle control, transcriptional regulation, cytoskeletal organization and developmental regulation [42].

### A nuclear transport Ran-GTPase is regulated by Pga1 signaling

The protein Pc22g01260, a putative Ran small GTPase, was in low abundance in strain Wis54-1255 with respect to all other strains/conditions, and was highly abundant (2.39-fold change) in the ∆*pga1* strain. Ran GTPases provide energy for the import–export transport through the nuclear pore by creating a Ran-GDP/Ran-GTP gradient across the membrane [43].

In a proteomic analysis of *Penicillium marneffei*, Chandler et al. [44] reported an increase of RanA, a Ran-GTPase, during the mould-to-yeast phase transition that occurs at 37 °C in this fungus. The increased levels of RanA correlate with the apparent co-ordination of cell division with nuclear division when the fungus grows at 37 °C, which leads to the generation of uninucleate yeast-like cells [45]. In addition, Flaherty and Dunkle [46] made a subtractive hybridization screening of ESTs expressed during dark-induced conidiation in *Exserohilum turcicum*, finding a gene encoding a Ran-GTPase protein which is highly expressed within the first 2 h of induction of conidiation. Here we found that Pc22g01260 is more abundant in the ∆*pga1* strain, which shows a hyperconidiating phenotype and develops a conidiation microcycle in submerged cultures [8], suggesting that the Pc22g01260 Ran-GTPase protein may function in the process of generation of uninucleate conidia in *P. chrysogenum*, a possibility that will be further investigated.

### A protein related to polarized growth is more abundant in the ∆*pga1* mutant

The Pc13g04170 protein, which is similar to the cell polarization protein Tea1p of *Schizosaccharomyces pombe*, is present in higher amount in strain ∆*pga1* (2.86-fold) and condition ∆*pga1* + cAMP (5.31-fold) as compared to strain Wis54-1255. Therefore we can conclude that it is negatively regulated by Pga1 and positively by cAMP. Tea1p regulates polarized growth in *S. pombe* [47]. A Tea1p homolog has been identified in *Aspergillus nidulans*, named TeaA, which is located predominantly at the Spitzenkörper, and the deletion of its encoding gene causes a zig-zag growth of the hyphae [48]. Absence of Pga1 causes hyperconidiation and a reduction of the diameter of the colony [10], but it is not known how Pga1 regulates apical extension of the hyphae, therefore the role of Pc13g04170 in *P. chrysogenum* remains to be elucidated.

### Proposed model for the Pga1-mediated signaling pathway and practical implications

Predicted interactions between the proteins identified in this work were analyzed in silico with the STRING v.10 [49] program (Additional file 2: Figure S1). A total of sixteen interactions were predicted, fourteen of which involve Pga1, and five are direct interactions between Pga1 and other identified proteins.

**Fig. 7:**
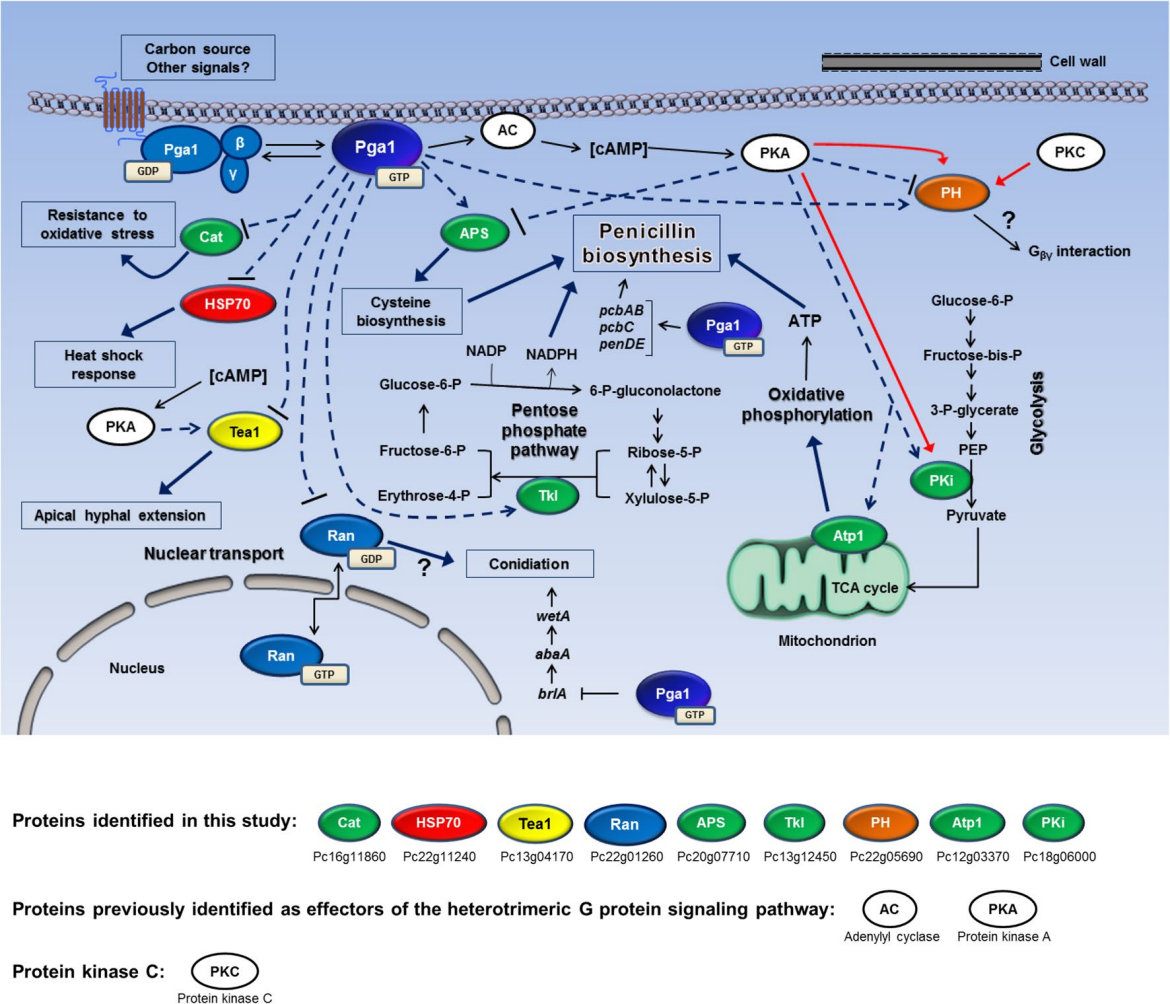
Model for the Pga1-mediated signal transduction pathway, showing the effectors AC and PKA previously reported in different fungi, along with newly identified effectors and proteins and their relation with the cellular processes regulated by Pga1.* Thin solid arrows* indicate steps in metabolic pathways, transport processes and established interactions of the fungal subgroup I Gα subunits. *Dotted lines* indicate positive (*arrows*) or negative (*bars*) effects on protein expression inferred from the results of this work. *Red solid arrows* indicate phosphorylation by PKA and PKC. *Thick solid arrows* indicate relation of identified proteins to cellular processes regulated by Pga1 signaling. *brlA* is the first gene of the central regulatory pathway of conidiogenesis, and is negatively regulated by Pga1 signaling [8]. *pcbAB*, *pcbC* and *penDE* are the three structural genes for penicillin biosynthesis, they are positively regulated by Pga1 signaling [10]

We have elaborated a model of the Pga1-mediated signaling pathway (Fig. 7) with the results obtained in this work, complemented with previous information on the cellular processes regulated by Pga1 signaling [7–10] and general established features of fungal subgroup I Gα protein-mediated signaling. Pga1 signaling controls morphogenic processes such as conidiation, which is repressed by Pga1 and triggered upon decrease of Pga1 activity [8]. In this work, we found that Pga1 signaling exerts control on the central catabolic pathways according to the cessation of vegetative growth of the hyphae occurring at the time of conidiation. Some proteins probably related to morphogenic processes were also identified as regulated by Pga1 (Tea1, Ran). Resistance to stress conditions is negatively regulated by Pga1 signaling [9], and we identified some effectors mediating this phenomenon (HSP90, CatR). Pga1 positively regulates penicillin biosynthesis [10], and we found that Pga1 signaling increases the abundance of enzymes participating in the biosynthesis of ATP, NADPH and cysteine, which are essential for high penicillin production [28, 30].

**Fig. 8:**
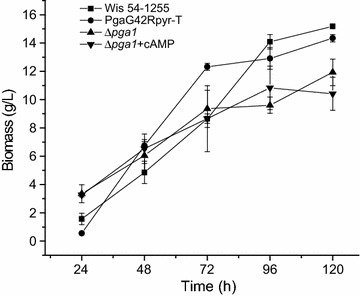
Biomass (dry weight) of the mycelium of the different strains and condition ∆*pga1* + cAMP obtained in flask cultivations with PMMY medium (see “Methods” section for details)

The activity of the Pga1-mediated signaling pathway can be manipulated through mutations of the *pga1* gene, as shown in this and previous works [8–10]. Therefore, the Pga1 pathway can be used as a target for the improvement of biotechnological processes in *P. chrysogenum*, including β-lactams biosynthesis, but also other processes that may be devised for this fungus, which has been the object of thorough bioengineering studies to adapt production processes to its physiology and metabolism. Manipulation of the Pga1-mediated pathway may lead to higher penicillin production yields [10], induce conidiation in submerged cultures [8], increase resistance to stress conditions [9], which may occur in bioreactors, and additionally, although poorly characterized yet, cause changes in the morphology of hyphae [8] and macroscopic morphology of mycelium. Manipulation of Pga1 activity can thus be a tool for the improvement of biotechnological industrial processes with *P. chrysogenum*. In this regard, it is important to consider how Pga1 activity affects the biomass in submerged cultures, so we performed a 120 h culture with the four conditions used in the proteomic analysis, and measured the produced biomass every 24 h (Fig. 8). The results indicated that there are significant differences in the biomass from 72 h of cultivation onwards; the lack of Pga1 activity (strain ∆*pga1*) caused a decrease in the produced biomass with respect to strains with an active Pga1, an effect that was not reversed by increasing intracellular cAMP levels. This is a point that must be taken into account when designing strategies involving the use of the *pga1* gene for improving the performance of *P. chrysogenum*. Nevertheless, the behavior of strains carrying mutations of the *pga1* gene may vary depending on the culture system, e.g. batch vs. continuous cultivation, the culture media and other culture parameters.

## Conclusions

The comparative proteomic analysis of *P. chrysogenum* strains with different levels of activity of the Pga1 Gα subunit has allowed us to identify probable new effectors that mediate Pga1 signaling, as well as proteins whose expression is regulated by Pga1 and participate in the cell response.

The use of the condition ∆*pga1* + cAMP in the proteome comparisons has been very useful to distinguish between cAMP-dependent (e.g. pyruvate kinase) and cAMP-independent (e.g. transketolase) regulation of protein expression by Pga1 signaling. In some cases there exist both types of regulation on the same protein, each having an opposite effect on the protein abundance (e.g. proteins Pc13g04170, Pc22g05690 and Pc22g17420).

Of special relevance is the identification of proteins and mechanisms involved in penicillin production, which can be utilized for strain improvement. Pga1 signaling positively regulates penicillin biosynthesis by increasing the expression of the three structural biosynthetic genes: *pcbAB*, *pcbC* and *penDE* [10]. Data from the proteomic analysis described here strongly suggest that, in addition, Pga1 signaling has an effect on penicillin biosynthesis by acting on primary metabolism pathways involved in cysteine, ATP and NADPH biosynthesis. We also found two proteins, Pc22g24530 (similar to dienelactone hydrolase) and Pc22g10220 (dnak-type molecular chaperone), which have also been identified in other proteomics studies associated to strains or conditions of high penicillin production [28, 32], and therefore they are worth further study to establish their exact role in penicillin biosynthesis.

This study has allowed us to elaborate a basic reference map for the functioning of the Pga1-mediated signal transduction pathway. Future work will focus on phosphoproteomic studies to detect effectors of the pathway through changes in phosphorylation, nuclear protein (phospho)proteomics to identify transcriptional regulators of the pathway, and further characterization of identified proteins, particularly the putative signal transducers.

Our study shows the potential of comparative proteomics and 2D-DIGE to analyze a signal transduction pathway using mutants with different levels of activity of the pathway and variations in the intracellular concentration of secondary messengers (induced increase of cAMP).

## Methods

### Strains and culture conditions

We used *P. chrysogenum* strains with different levels of Pga1 activity: Wis54-1255 (wild type Pga1), PgaG42Rpyr-T (carrying a dominant *pga1* ^G42R^ allele and expressing a constitutively active Pga1) [8] and ∆*pga1* (no Pga1 activity) [8]. We also used the ∆*pga1* strain with induced high intracellular cAMP concentrations (condition ∆*pga1* + cAMP), in order to differentiate cAMP-mediated and cAMP-independent Pga1 signaling. The strains/conditions are described in Table 3.

**Table 3:** *Penicillium chrysogenum* strains used for the proteomic analysis, their genotypes and Pga1 activities

Strain	Genotype/Pga1 function	Reference
Wis54-1255^a^	Wild type *pga1*, normal Pga1 function	ATCC 28089
PgaG42Rpyr-T	*pga1* ^*G42R*^, constitutively active Pga1 subunit	García-Rico et al. [8, 10]
Δ*pga1*	*pga1* gene deleted, absent Pga1 subunit	García-Rico et al. [8, 10]
Δ*pga1* + cAMP^b^	*pga1* gene deleted, absent Pga1 subunit (intracellular cAMP levels increased)^b^	García-Rico et al. [8, 10]

^a^Also known as Wisconsin 54-1255

^b^db-cAMP and theophylline were used to increase the level of intracellular cAMP

All strains were grown on Power solid medium [50] at 28 °C for 5–6 days. Conidia were collected by shaking with a solution of Tween 80 (0.01 %). The PMMY medium ([g/l]: glucose 40, NaNO_3_ 3, yeast extract 2, NaCl 0.5, MgSO_4_ 0.5, FeSO_4_ 0.01) was used to obtain mycelium from submerged cultures. The cultures were performed in 500 ml flasks with 100 ml of PMMY medium; they were inoculated with a final concentration of 10^7^ spores/ml and incubated for 72 h at 250 rpm and 25 °C. The mycelium was then collected by filtration, washed with 0.9 % NaCl, and stored at −80 °C until required.

### Quantification of cAMP and induction of high cAMP intracellular concentrations in strain Δ*pga1*

Quantification of cAMP concentration in the mycelium of the different strains/conditions was performed as previously described [8].

The strain ∆*pga1* has naturally low cAMP intracellular concentrations [8, 9]. Increasing cAMP concentration in this strain was performed as previously described [8], using db-cAMP (0.1 mM) and theophylline (0.05 mM) in the culture medium (PMMY). This condition was named Δ*pga1* + cAMP.

### Determination of mycelium biomass in submerged culture

The three strains and condition ∆*pga1* + cAMP (described in Table 3) were grown on solid medium, inoculated in 250 ml flasks with 50 ml of PMMY medium and incubated as described in the “Strains and culture conditions” section. Three replicates were used for each strain/condition and time point. Every 24 h the mycelium was collected from the flasks by filtration, washed with distilled water and dried in an oven until the dry weight remained constant.

### Obtention of intracellular protein extracts from *Penicillium chrysogenum*

We followed the method described by Jami et al. [28] with modifications. Mycelium from the different strains (stored at −80 °C) was ground in a mortar with liquid nitrogen, and 2 g of the resulting powder was mixed with 10 ml of 10 mM potassium phosphate pH 7.4 containing 100 μl of protease inhibitor cocktail for fungal and yeast cells (Sigma-Aldrich). The mixture was sonicated for 10 min on ice, and then shaken for 2 h at 4 °C. The protein extract thus obtained was centrifuged at 15,300×*g* for 10 min, and proteins in the supernatant were precipitated overnight at −20 °C with a solution of 20 % trichloroacetic acid in acetone and 0.14 % dithiothreitol. Proteins were pelleted by centrifugation at 25,900×*g* for 15 min at 4 °C. The pellet was washed twice with 100 % acetone and then once with 80 % acetone, and the final precipitate was solubilized with 1 ml of rehydration solution (7 M urea, 2 M thiourea, 4 % CHAPS, 30 mM Tris). The insoluble fraction was discarded by centrifugation at 18,000×*g* for 5 min. The protein concentration in the supernatant was determined with the Protein Assay Dye Reagent Concentrate (Bio-Rad) following the manufacturer’s indications.

### Experimental design of 2D-DIGE

We followed an experimental scheme previously reported by Vera-Estrella et al. [21]. A total of eight gels were run, covering the six possible combinations between the four conditions plus two repetitions for pairs PgaG42Rpyr-T *vs*. ∆*pga1*, and Wis54-1255 *vs*. ∆*pga1* + cAMP (Table 4). For each gel, proteins from one condition were labeled with Cy3 and those from another condition with Cy5, and a normalization mixture of samples of the four conditions was labeled with Cy2 and used as the internal standard to allow gel to gel comparisons.

**Table 4:** Experimental design for 2D-DIGE analysis of proteins extracted from mycelium of different *P. chrysogenum* strains

	Cy3 (50 µg of protein)	Cy5 (50 µg of protein)	Cy2 (50 µg total protein, 3.125 µg each sample)
Gel 1	W1	G1	W1 + W2 + W3 + W4 + Δ1 + Δ2 + Δ3 + Δ4 + Δ*1 + Δ*2 + Δ*3 + Δ*4 + G1 + G2 + G3 + G4
Gel 2	Δ1	W2	W1 + W2 + W3 + W4 + Δ1 + Δ2 + Δ3 + Δ4 + Δ*1 + Δ*2 + Δ*3 + Δ*4 + G1 + G2 + G3 + G4
Gel 3	W3	Δ*1	W1 + W2 + W3 + W4 + Δ1 + Δ2 + Δ3 + Δ4 + Δ*1 + Δ*2 + Δ*3 + Δ*4 + G1 + G2 + G3 + G4
Gel 4	Δ2	G2	W1 + W2 + W3 + W4 + Δ1 + Δ2 + Δ3 + Δ4 + Δ*1 + Δ*2 + Δ*3 + Δ*4 + G1 + G2 + G3 + G4
Gel 5	Δ*2	Δ3	W1 + W2 + W3 + W4 + Δ1 + Δ2 + Δ3 + Δ4 + Δ*1 + Δ*2 + Δ*3 + Δ*4 + G1 + G2 + G3 + G4
Gel 6	G3	Δ*3	W1 + W2 + W3 + W4 + Δ1 + Δ2 + Δ3 + Δ4 + Δ*1 + Δ*2 + Δ*3 + Δ*4 + G1 + G2 + G3 + G4
Gel 7	Δ*4	W4	W1 + W2 + W3 + W4 + Δ1 + Δ2 + Δ3 + Δ4 + Δ*1 + Δ*2 + Δ*3 + Δ*4 + G1 + G2 + G3 + G4
Gel 8	G4	Δ4	W1 + W2 + W3 + W4 + Δ1 + Δ2 + Δ3 + Δ4 + Δ*1 + Δ*2 + Δ*3 + Δ*4 + G1 + G2 + G3 + G4

Strains: W = Wis54-1255, G = PgaG42Rpyr-T, Δ = Δ*pga1*, Δ* = Δ*pga1* + cAMP

The number after the letter of the strain is the sample number; each sample comes from mycelium of an independent culture

The 2D-DIGE technique has some advantages over conventional 2D-PAGE. The use of three different fluorophores (CyDye DIGE fluors) permits running the two samples to be compared in the same gel, along with a third normalization sample consisting of a mixture of all the conditions under study (four in our case). This allows multi-plexing and reduces the number of gels necessary to compare all conditions in pairs, and also decreases variability and provides more reliable quantitative results due to the presence of the normalization sample in each gel [51, 52].

### Preparation of samples and carrying out of 2D-DIGE

Four biological replicas (independent cultures) of each of the four conditions (strains Wis54-1255, PgaG42Rpyr-T, ∆*pga1*, and condition ∆*pga1* + cAMP) were used, giving a total of 16 samples. The labeling of proteins from each sample with the corresponding Cy-dye was performed according to the manufacturer’s instructions (DIGE minimal labeling protocol, GE Life Sciences), using 75 μg of protein. The labeling reactions were stopped by adding 1 μl of 10 mM lysine and incubating for 10 min.

Fifty micrograms of labeled protein from two of the samples (one with Cy3 and one with Cy5 according to Table 4), plus 50 μg of the Cy2-labeled internal standard normalization mixture (containing 3.125 μg of each of the 16 samples) were mixed together in a final volume of 300 μl of rehydration buffer [7 M urea, 2 M thiourea, 2 % (w/v) CHAPS, 0.5 % DTT, 0.5 % ampholytes pH 3–10 (Bio-Rad)]. Eight such mixtures were carried out, one for each of the gels to be run. These mixtures were loaded into individual wells on an IEF focusing tray (Bio-Rad), and then the IPG ReadyStrips of 17 cm with a lineal pH range 3–10 (Bio-Rad) were placed onto the tray gel side down. Active rehydration was carried out at 20 °C in the dark, for 16 h at 50 V. Isoelectric focusing was performed with the following program: 250 V for 15 min, a 250 V to 4000 V gradient for 2.5 h, a 4000 V to 15 kV gradient for 12 h, in a PROTEAN^®^ IEFCell (Bio-Rad). Once the isoelectric focusing was completed, the IPG ReadyStrips were equilibrated for 15 min with the following solution: 50 mM Tris HCl (pH 8.8), 6 M urea, 30 % (v/v) glycerol, 2 % (w/v) SDS, 0.002 % bromophenol blue, 2 % (w/v) DTT; and then for another 15 min with the same solution substituting the DTT by 2.5 % (w/v) iodoacetamide.

The second dimension SDS-PAGE (12 % acrylamide) was performed in an Ettan Dalt Six electrophoresis system (GE Life Sciences), using low fluorescence glass plates with one side of each pair coated with bind-silane (GE Life Sciences). Gels were run overnight at 12 V and 25 °C. The gels were then imaged using a Typhoon 9400 scanner (GE Life Sciences), using the emission band-pass wavelengths corresponding to each of the fluorophores: Cy2 (520/40 nm, laser blue 488 nm), Cy3 (580/30 nm, laser green 532 nm) and Cy5 (670/30 nm, laser red 633 nm), with 100 μm of resolution and 600 V photomultiplier tube voltage.

### Analysis of the images and extraction of the spots

The analysis of the images from the scanned gels was done with the DeCyder 2D software v6.5 (GE Healthcare). First we used the DIA (Differential In-gel Analysis) module to detect all the spots in the eight gels and quantify the fluorescence corresponding to the different conditions. We then used the BVA (biological variation analysis) module to compare the changes in abundance between the different conditions. Only the spots showing statistically significant (ANOVA *p* value ≤0.05) changes in abundance above ±1.5-fold between two conditions were considered to proceed with their analysis. The spots were excised from the gels with the robotic Ettan Spot Handling Workstation (GE Life Sciences) using the spot maps obtained from the DeCyder software.

### Identification of proteins by mass spectrometry (LC–MS/MS)

Trypsin digestion was performed as previously described [53] with some modifications. The plugs (spots extracted from the gels) were washed and destained in 0.5 ml of a methanol/acetic acid (1:1) solution overnight, and then dehydrated in 100 μl acetonitrile for 5 min. The supernatant was removed, and the plugs were rehydrated in a 20 μl solution of trypsin (20 ng/ml) for 10 min, until the solution was absorbed. Then the plugs were covered with a solution of 50 mM NH_4_HCO_3_ and incubated at 37 °C overnight, after which another 20 μl of the same solution were added, and the plugs were maintained for 10 min at room temperature. The samples were centrifuged and the supernatants (containing the peptides resulting from the trypsin digestion) were transferred to 0.5 ml tubes. A second extraction of trypsin-digested peptides from the plugs was performed by adding 20 μl of a solution of 5 % formic acid in 50 % acetonitrile, and incubating at room temperature for 10 min. The plugs were then centrifuged for 30 s, and the supernatants were combined with those previously obtained. The solution containing the peptides was desalted by extraction in solid phase with C18 Zip-Tips (Millipore), and then the peptides were eluted with 15 μl of a solution 1 % formic acid in 50 % acetonitrile.

The peptides were then analyzed by nano-LC–MS/MS, using an auto sampler Finnigan MicroAS and a Surveyor MS pumping system coupled to a LTQ-Orbitrap (Thermo). Forty microliters of each sample were injected into a C18 precolumn (Symmetry C18 NanoEase Trap Column 5 μm, Waters) with a 3 μl/min flux of 0.1 % formic acid in 5 % acetonitrile for 15 min. Then the peptides were eluted with a 300 nl/min flux of a 5–35 % gradient of solvent B (0.1 % formic acid in 100 % acetonitrile) for 60 min in a BioBasic C18 PicoFrit column (PFC7515-BI-10, New Objective) and automatically loaded into the ESI-LTQ. Data acquisition was performed with the Xcalibur program. A Fourier transform (FT) full scan MS spectra was acquired with the Orbitrap detector in a m/z range of 300–1800, with resolving power set at 30,000 (400 m/z). The five peaks with higher intensity were analyzed in the ion trap by collision-induced dissociation (CID). The MS/MS raw spectra data were converted to DTA files using Thermo Electron Bioworks 3.2, and then analyzed by means of TurboSEQUEST (Thermo Fisher Scientific).

Tandem mass spectra were analyzed using Mascot (Matrix Science, version 2.3.02). Mascot was set up to search the *Penicillium chrysogenum* (current name: *Penicillium rubens*), taxonomy ID 500485, database (25,676 entries) assuming the digestion enzyme trypsin. Mascot was searched with a fragment ion mass tolerance of 0.60 Da and a parent ion tolerance of 10 ppm. Carbamidomethyl of cysteine was specified in Mascot as a fixed modification. Oxidation of methionine and phosphorylation of serine, threonine and tyrosine were specified in Mascot as variable modifications.

### Validation of the identification of proteins

Validation of protein identification from MS/MS was carried out with the program Scaffold_4.0.5 (Proteome Software Inc.). Peptide identifications were accepted if they could be established at greater than 95 % probability by the peptide prophet algorithm [54] with Scaffold delta-mass correction. Confirmed identification of a protein was considered when the probability was above 99 % and at least two peptides from the protein were positively identified.

### Prediction of phosphorylation sites

The programs pkaPS [22] and NetPhosK 1.0 of the NetPhos Server [55] were used to find putative phosphorylation sites by different protein kinases in the amino acid sequence of some of the proteins; these sites were compared with the phosphorylated peptides found by LC–MS/MS (Table 2).

### Prediction of protein–protein interactions

We used the program STRING v10 (string-db.org) to predict physical or functional interactions between the proteins whose expression is under control of Pga1 signaling.

## Additional files

10.1186/s12934-016-0564-x Additional information for protein identification by MS, showing the sequence of exclusive unique identified peptides and the chemical modifications present in them.

10.1186/s12934-016-0564-x In silico interactome of the proteins identified in this work. The analysis was performed with the program STRING v10.

## Abbreviations

2D-DIGE: two dimensional differential in-gel electrophoresis
LC–MS/MS: liquid chromatography-tandem mass spectrometry
db-cAMP: N6,2′-*O*-dibutyryladenosine 3′, 5′-cyclic monophosphate
