# Supplementary material for: Proteomic analysis of the signaling pathway mediated by the heterotrimeric Gα protein Pga1 of Penicillium chrysogenum
Source: Microb Cell Fact. 2016 Oct 6;15:173. doi: 10.1186/s12934-016-0564-x (PMC5053351; doi:10.1186/s12934-016-0564-x)
Supplement: Supplementary file 2 — 10.1186/s12934-016-0564-x In silico interactome of the proteins identified in this work. The analysis was performed with the program STRING v10. [file 12934_2016_564_MOESM2_ESM.docx]

**Additional file 2**. **Fig. S1**. *In silico* interactome of the proteins identified in this work. The analysis was performed with the program STRING v.10. The colours of the lines indicate the type of interaction, which is shown in the column at the right of the diagram. There are two direct interactions between Pga1 and the Pc12g05640 protein (putative HSP90 heat shock protein similar to *Aspergillus niger* SspB). In humans, a similar interaction has been previously reported between the Gα subunit of the heterotrimeric G12 protein and a HSP90, and this interaction was demonstrated to be important for Gα-mediated signaling [Vaiskunaite et al. J Biol Chem. 2001; 276: 46088-46093]. Direct interactions with Pga1 were also predicted for two putative ribosomal proteins and two probable components of the mitochondrial ATP synthase.
